# Modifying Alcohol Consumption to Reduce Obesity: A Randomized Controlled Feasibility Study of a Complex Community-based Intervention for Men

**DOI:** 10.1093/alcalc/agx067

**Published:** 2017-09-18

**Authors:** Linda Irvine, Iain K Crombie, Kathryn B Cunningham, Brian Williams, Falko F Sniehotta, John Norrie, Ambrose J Melson, Claire Jones, Peter Rice, Peter W Slane, Marcus Achison, Andrew McKenzie, Elena D Dimova, Sheila Allan

**Affiliations:** 1 Division of Population Health Sciences, School of Medicine, University of Dundee, The Mackenzie Building, Kirsty Semple Way, Dundee, UK; 2 School of Health & Social Care, Edinburgh Napier University, Sighthill Campus, Sighthill Court, Edinburgh, UK; 3 Institute of Health and Society, Medical Faculty, University of Newcastle, UK; 4 Edinburgh Clinical Trials Unit (ECTU), University of Edinburgh, No. 9, Bioquarter, Medical School, Teviot Place, Edinburgh, UK; 5 Institute of Health and Wellbeing, University of Glasgow, Mental Health & Wellbeing Academic Centre, Gartnavel Royal Hospital, 1055 Great Western Road, Glasgow, UK; 6 Health Informatics Centre, University of Dundee, Ninewells Hospital & Medical School, Dundee, UK; 7 Division of Neuroscience, School of Medicine, University of Dundee, Dundee, UK; 8 Erskine Practice, Arthurstone Medical Centre, Dundee, UK; 9 Faculty of Health Sciences and Sport, University of Stirling, Stirling, UK; 10 Dundee City Council, Community Health Inequalities Manager, Mitchell Street Centre, Dundee, UK

## Abstract

**Objectives:**

Being obese and drinking more than 14 units of alcohol per week places men at very high risk of developing liver disease. This study assessed the feasibility of a trial to reduce alcohol consumption. It tested the recruitment strategy, engagement with the intervention, retention and study acceptability.

**Methods:**

Men aged 35–64 years who drank >21 units of alcohol per week and had a BMI > 30 were recruited by two methods: from GP patient registers and by community outreach. The intervention was delivered by a face to face session followed by a series of text messages. Trained lay people (Study Coordinators) delivered the face to face session. Participants were followed up for 5 months from baseline to measure weekly alcohol consumption and BMI.

**Results:**

The recruitment target of 60 was exceeded, with 69 men recruited and randomized. At baseline, almost all the participants (95%) exceeded the threshold for a 19-fold increase in the risk of dying from liver disease. The intervention was delivered with high fidelity. A very high follow-up rate was achieved (98%) and the outcomes for the full trial were measured. Process evaluation showed that participants responded as intended to key steps in the behaviour change strategy. The acceptability of the study methods was high: e.g. 80% of men would recommend the study to others.

**Conclusions:**

This feasibility study identified a group at high risk of liver disease. It showed that a full trial could be conducted to test the effectiveness and cost-effectiveness of the intervention.

**Trial registration:**

Current controlled trials: ISRCTN55309164.

**Trial funding:**

National Institute for Health Research Health Technology Assessment (NIHR HTA).

**Short summary:**

This feasibility study recruited 69 men at high risk of developing liver disease. The novel intervention, to reduce alcohol consumption through the motivation of weight loss, was well received. A very high follow-up rate was achieved. Process evaluation showed that participants engaged with key components of the behaviour change strategy.

## INTRODUCTION

Men who drink in excess of 14 units of alcohol per week and are obese have a 19-fold higher risk of dying from liver disease compared to normal weight men who do not drink alcohol (Hart *et al.*, 2010). Being obese and drinking heavily each increase the risk of liver disease, but a combination of the two has a supra-additive effect on risk (Hart *et al.*, 2010; Mahli and Hellerbrand, 2016). Heavy alcohol consumption is also associated with an increased risk of obesity (Arif and Rohrer, 2005; Traversy and Chaput, 2015) and heavy drinking leads to overeating (Lloyd-Richardson *et al.*, 2008), increasing further the potential for weight gain. Tackling the two problems simultaneously could make a significant contribution to improving public health (Lavin *et al.*, 2016). The National Institute for Health Research (NIHR) Health Technology Assessment (HTA) commissioned a feasibility study to develop an intervention to reduce alcohol consumption among obese men through the motivation of weight loss. The HTA brief specified that an established alcohol brief intervention (ABI) should be used as the comparator.

Intervening with men who are obese and drink above recommended levels is challenging. Both obesity and alcohol consumption are sensitive issues and recruiting men to a study which addresses these two behaviours is likely to be difficult. Attrition is a problem in ABIs studies (Kaner *et al.*, 2007), and is typically high in weight loss studies (Douketis *et al.*, 2005), and challenges the external validity of their findings. In addition, many men may not think that drinking above recommended levels is harmful to their health and may be reluctant to change their drinking.

This paper reports on the feasibility of a novel, community-based, gender-sensitive alcohol intervention for men who are obese. Guidance on developing and evaluating complex interventions recommends that feasibility testing should always take place prior to evaluation of effectiveness (Craig *et al.*, 2008) so that weaknesses in methodology can be identified and rectified (Lancaster *et al.*, 2004; Bowen *et al.*, 2009; Arain *et al.*, 2010; Leon *et al.*, 2011). The main aims of the feasibility study were: to determine the best ways to recruit and retain men who are obese in a study intended to reduce heavy drinking; to design a novel intervention and assess the fidelity of its delivery; to evaluate whether the intervention engages the participants; to test whether the outcomes to be used in a full randomized controlled trial can be measured; and to measure the acceptability of the study methods.

## METHODS AND PROCEDURES

The study received ethical approval from the East of Scotland Research Ethics Service (Reference 14/ES/0050). The study is registered as: ISRCTN55309164.

### Participants

Participants were men aged 35–64 years who drank >21 UK units of alcohol per week and had a BMI > 30 kg/m^2^. Exclusion criteria were men: who were attending alcohol problem services; who were attending weight management classes/services; or who would not be contactable by mobile phone during the study. The intention was recruit 60 participants. Although a formal sample size calculation was not needed for the feasibility study, the intended total was judged to be sufficient to test the feasibility of recruitment and retention, the extent of engagement with the intervention and the acceptability of the study methods.

### Recruitment

Two recruitment strategies were evaluated: recruitment from primary care registers and time-space sampling (TSS) (Semaan, 2010), a community outreach method. Men were recruited from March to June 2015. Half of the participants were recruited from the practice lists of three general practices. Potential participants (selected on age and having a recorded BMI > 30 kg/m^2^) received a letter from their GP inviting them to take part in the study. An opt-out strategy was used and researchers contacted by phone individuals who did not opt out of the study ~2 weeks after the GP letter was sent. The remaining 50% of participants were recruited by TSS (Semaan, 2010) from a variety of venues including a town centre, workplaces, community groups, football grounds and a golf club. Men identified by the recruitment strategies were screened by a telephone interview and those who fitted the entry criteria and were willing to take part gave consent by text message.

### Randomization, allocation concealment and blinding

Randomization was carried out using the secure remote web-based system provided by the Tayside Clinical Trials Unit. Randomization was stratified by the recruitment method and restricted using block sizes of randomly varying lengths. Participants’ data (mobile phone number, study ID number and preferred first name) were entered into the web-based randomization system which assigned men to treatment arms. The researcher who recruited the men had no access to the randomization system. The researchers who conducted the baseline and follow-up interviews were unaware of treatment group.

### Intervention

The intervention was designed to be delivered in two phases, which comprised a face to face session followed by a series of text messages. The behaviour change strategy was based on the Health Action Process Approach (HAPA) (Schwarzer, 2008). The strategy also used techniques shown to be effective with obese adults in weight loss interventions (Dombrowski *et al.*, 2012).

The face to face session was delivered by trained lay people. Lay people can have an important role in the prevention of chronic disease, promoting positive healthful behaviours among their peers (Brownstein *et al.*, 2005). The face to face session was intended to increase intentions to drink less through the motivation of weight loss. The session capitalized on the measurement of alcohol consumption and BMI, as feedback of information on current behaviour is a common technique in ABIs (Kaner *et al.*, 2007). Thus, the intervention gave men the opportunity to calculate for themselves the units of alcohol and the calories in the alcohol they consumed. The men also plotted their height and weight on a BMI chart. Together these measurements and calculations illustrated the intended logic of the intervention; that reducing alcohol consumption could result in weight loss. This was reinforced by a discussion of the way alcohol increased food intake (e.g. by increased snacking) and the benefits that the men would enjoy if they lost weight.

The second phase of the intervention focussed on taking action to change behaviour, the volitional component of HAPA (Schwarzer, 2008). A series of 95 text messages were delivered over a period of 2 months by a computer system described elsewhere (Irvine *et al.*, 2012). The messages reinforced the topics discussed during the face to face session to provide a platform for the setting of goals to reduce alcohol consumption and the creation of action plans for drinking less. Coping strategies, relapse prevention and maintenance of the new behaviour were introduced and reinforced.

The HTA brief specified that an established ABI should be used as the comparator. The comparator was an ABI delivered in one face to face session by trained lay people. It was based on an ABI used in the Screening and Intervention Programme for Sensible drinking (SIPS) (Kaner *et al.*, 2013). It used the SIPS Simple Structured Advice Intervention Tool to give advice on the risks of alcohol-related harm and the benefits of cutting down. Participants were encouraged to make plans to reduce their drinking. Men in the comparator group received five text messages during the 2 months following the face to face session. These texts were designed to increase retention only, e.g. by prompting men to report changes in their address or phone number. The texts did not mention alcohol, weight or behaviour change.

### Training of the lay people

Bespoke training programmes were developed for the lay people. To prevent contamination, lay people were assigned to deliver either the intervention or comparator packages. They were trained separately and were unaware of whether they were delivering the intervention or the comparator. Six lay people were required (three intervention and three comparator), but 12 people were selected at interview to be trained. The final selection of candidates took place after the training was complete. This method of over-selection allowed for drop-out during the training period and enabled selection of the most competent individuals.

All candidates were trained in measuring height, weight, and obtaining an accurate history of drinking. The intervention group lay people also received training in calculating BMI and in Motivational Interviewing (MI) based skills (Miller and Moyers, 2006). The comparator group lay people were trained to deliver the established SIPS brief intervention (Kaner *et al.*, 2013) using materials provided on the SIPS website. Separate manuals, which provided a step by step guide for the delivery of their session, were prepared for intervention and comparator groups. These were based on current guidance for training manuals (British Psychology Society Division of Health Psychology team: *et al.*, 2008; Dixon and Johnston, 2010).

### Fidelity of delivery

To assess the extent to which the intervention was delivered as intended (Borrelli, 2011; Leeuw *et al.*, 2009), the face to face sessions were audio-recorded and assessed for adherence (components of the intervention were covered) and competence (delivered to a high standard). Separate task lists to monitor adherence were used for intervention and comparator groups. Performance at these tasks was assessed by a research psychologist (E.D.D.). The lay people who delivered the intervention were also assessed on their competence using relevant items from the Behaviour Change Counselling Index (BECCI) (Lane, 2002). The items covered were: showing empathy, being sensitive to the participants’ views, asking open questions and summarizing at the end of the session. Performance at each intervention session was assessed (by E.D.D.) and constructive feedback was given. Fidelity of delivery of the text message component of the intervention was assessed by tracking electronically whether the messages were delivered to the participants’ mobile phones and by monitoring responses received from the participants using methods described elsewhere (Irvine *et al.*, 2012).

### Baseline and outcome data collection

Baseline height, weight and alcohol consumption were measured at the face to face session. Socio-demographic data including education, employment, Scottish Index of Multiple Deprivation (SIMD), an area-based measure of social deprivation based on postcode (Office of the Chief Statistician, 2004), and marital status were also collected. Weight was measured by Seca 813 medical scales. Height was measured using a portable Seca 213 stadiometer with men in stockinged feet. The alcohol Time Line Followback (TLFB) questionnaire (Sobell, 2003) was used to measure alcohol consumption accurately over the previous 28 days. The Fast Alcohol Screening Test (FAST) (Health Development Agency and University of Wales College of Medicine, 2002) was administered to identify hazardous drinkers. A questionnaire was administered at the end of this session to assess acceptability of the study methods. Additionally, the face to face sessions were audio-recorded to monitor adherence to and competence at delivering the intervention. Adherence was assessed against a checklist of key tasks and competence by items from the BECCI (Lane, 2002).

Five months after baseline (between August and November 2015), men were invited to attend the follow-up face to face session, at which weight and alcohol consumption were measured. Questionnaires were also administered to assess hazardous drinking, drinking refusal skills, recall of the intervention and acceptability of the study methods. Knowledge of alcohol units, BMI and of calories in alcoholic drinks was assessed. Experience of attempting to reduce alcohol intake was explored across several questions, which asked about awareness of harms, plans to reduce drinking, and perceived benefits of cutting down.

## RESULTS

### Recruitment

**Fig. 1.**
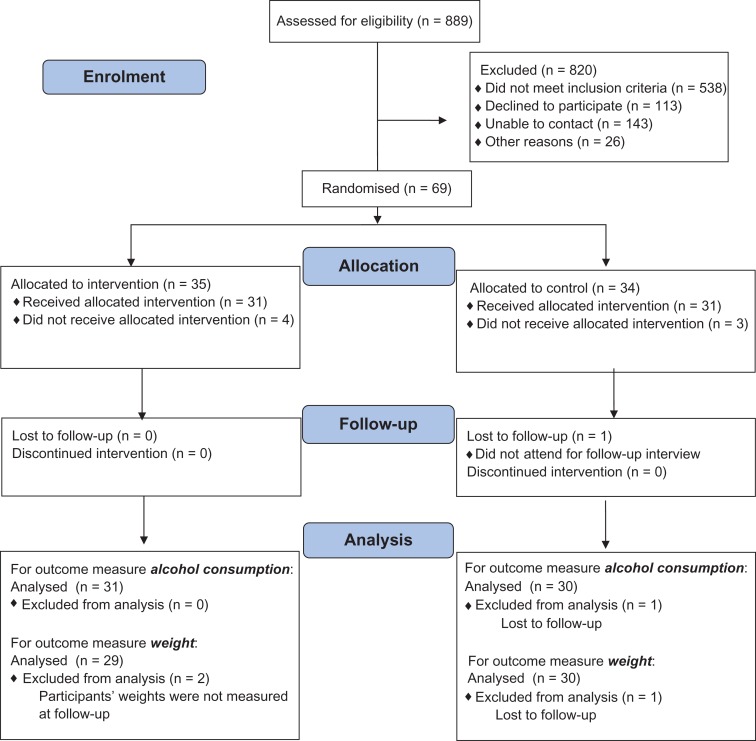
Study flow diagram.

A total of 889 men were assessed for eligibility to take part in the study (Fig. 1). Overall, 200 men aged 35–64 years with a recorded BMI > 30 kg/m^2^ were randomly selected from each of the three GP registers. Of these 167 were screened out by their GP and 14 opted out. Of the 419 to be contacted, many men (44%) reported drinking less than the 21 units per week required for the entry criterion. Of the 470 men approached by TSS just over half (53%) reported drinking <21 units per week. Recruitment by TSS proved onerous, with an average of one man recruited for every 11 fieldwork visits made. The recruitment target of 60 participants was exceeded, and 69 men were recruited and randomized. Three of these men could not be contacted to make an appointment for the face to face session, and four withdrew when contacted. The remaining 62 men received either the intervention or comparator packages.

### Baseline data

The mean age of the participants was 52.5 years, the majority (71%) lived with a partner and 77% were in employment (Table 1). More than 40% of the men lived in the most disadvantaged quintile (as measured by the Scottish Index of Multiple Deprivation) (Office of the Chief Statistician, 2004). Almost all of the men (92%) were classified as hazardous drinkers using the FAST (Health Development Agency and University of Wales College of Medicine, 2002) (Table 1). Mean weekly alcohol consumption was 47 units per week, well above the inclusion criterion of >21 units per week. There was a marked imbalance between treatment groups in alcohol consumption, with a mean consumption of 53.3 units in the comparator group, and 41.1 units in the intervention group. Mean BMI was 35.7 kg/m^2^ and was similar in both groups, again much higher than the entry criterion of 30 kg/m^2^.

**Table 1.** Baseline characteristics of participants by treatment group

Factor	Comparator group, *N* = 31	Intervention group, *N* = 31	Total, *N* = 62
Marital status (*n*, %)			
Single	9 (29.0)	7 (22.6)	16 (25.8)
Married/lives with a partner	22 (71.0)	24 (77.4)	46 (74.2)
Scottish Index of Multiple Deprivation (SIMD) quintile (*n*, %)			
1–2 (Most disadvantaged)	13 (41.9)	14 (45.2)	27 (43.5)
3–5 (Least disadvantaged)	18 (58.1)	17 (54.8)	35 (56.5)
Employment status (*n*, %)			
Employed	26 (83.9)	22 (71.0)	48 (77.4)
Not in employment	5 (16.1)	9 (29.0)	14 (22.6)
Hazardous drinkers (positive FAST) (*n*, %)	30 (96.8)	27 (87.1)	57 (91.9)
Mean weekly consumption (mean units, SD)	53.3 (40.7)	41.1 (31.9)	47.2 (36.8)
Mean number of drinking days in previous 28 days (mean, SD)	14.7 (7.3)	15.2 (6.3)	15.0 (6.8)
Mean number of binge drinking days in previous 28 days (>8 units in one session) (mean, SD)	9.9 (6.5)	8.7 (5.5)	9.3 (6.0)
Mean BMI (SD)	35.5 (3.9)	35.9 (5.4)	35.7 (4.7)

### Fidelity of delivery of the face to face session

The audio recordings of the sessions showed that adherence to all tasks was very high for all of the lay people: only one item was missed and that was on a single occasion. Competence for the intervention group Study Coordinators, as assessed by components of the BECCI, was mostly satisfactory. The maximum possible score was 4.0. The mean score was high on some items such as displaying empathy (3.62) and sensitivity to the participant’s concerns (3.59). The more challenging items were encouraging discussion of current drinking (2.9), and providing summaries at the end of the sessions (3.17).

### Fidelity of delivery of the text messages

The intervention package included 95 SMS text messages. Thus, a total of 2945 messages were sent to the 31 participants during the intervention period. Of these, 2887 messages (98%) were delivered to the participants’ telephones. The remaining 58 messages were recorded as undelivered. In total, 22 men failed to receive one or more messages. The number of undelivered messages per participant ranged from one to seven with a median two messages missed. None of the participants missed consecutive messages.

Engagement with the study was assessed by the responses to the text messages. Responses were received from all but two of the participants (94%). A total of 456 messages were received from the remaining 29 men. The number of responses per participant ranged from 0 to 41 (mean 14.7, median 12). More than 60% of the men responded more than 10 times. However, there was marked variation. Four men responded more than 35 times, while seven responded on fewer than five occasions.

Comprehension and engagement with key components of the intervention were assessed by reviewing the content of responses received. For key steps in the behaviour change sequence, text messages requested a response to specific questions (Table 2). The nature of their responses showed that men had understood the text messages and had put thought into their replies. For example, in response to a question on how drinking influences what you eat, men gave examples of making high calorie food choices such as kebabs and pizza. The question on the main benefit of changing their current drinking pattern, elicited responses about losing weight, being more active and improved health.

**Table 2.** Responses to key components of the behaviour change strategy

Component of the behaviour change strategy addressed by text message question	Number of responses^a^	Examples of text message responses received from participants
Self-monitoring of alcohol consumption	17	5 pints And 7 nips I’ve done great this week
Awareness that drinking encourages unhealthy eating	23	I eat a lot of junk food while having a can of beer in the house
Perceived benefits of drinking less	21	To stave off periods of gout, lose weight, feel generally healthier
Awareness of harmful effects of obesity	21	I struggle on the golf course after 1st 9
Who would support drinking less	17	My kids…they would tell me I’ll live longer if I cut back
Goal setting	17	No drinking mid week would be a goal
Action planning	13	I’ve got a plan. Instead of buying 75cl bottles I’m going to buy 37.5’s instead
Perceived benefits of changing current drinking	19	Getting my health back and getting back into my 32 jeans
Coping planning	9	I would go to my workshop or gym and try to keep myself busy
Reported actual benefits of drinking less at the end of the intervention period	12	Yes, feeling fresher in the mornings and getting into work sharp.

^a^Maximum number of responses = 28.

### Acceptability of the study methods

The acceptability of the study to participants was assessed using indirect questions such as whether they discussed the study with anyone and whether they would recommend the study to others. Acceptability ranged from 70 to 90%, depending on question phrasing, with acceptability levels being similar for intervention and comparator groups. The highest level of acceptability reported (90%) was for the perceived benefit of taking part in the study. Common specific benefits were increased awareness of personal alcohol consumption (*n* = 25) and having stopped or reduced drinking (*n* = 16). Most participants (>80%) would recommend the study to others. The men were most likely to recommend the study to partners (*n* = 31) and to friends and colleagues (*n* = 22).

### Retention of participants

Participants were followed up 5 months after recruitment, which was 3 months after the text message component of the intervention was delivered. Of the 62 men who attended the baseline face to face session, 61 (98%) were interviewed at follow-up (Fig. 1). Overall, 59 men (95%) attended a session at which their weight was measured and questionnaires were completed. The remaining two men were interviewed by telephone at which they gave a self-reported weight. The weights reported by these men were not included in the analyses.

### Alcohol consumption and weight at follow-up

Mean weekly alcohol consumption and BMI will be the primary outcomes for a randomized controlled trial, so the main purpose of measuring these in the feasibility study was to ensure they could be measured at baseline and follow-up.

In keeping with guidance for feasibility studies, no statistical tests were carried out (Leon *et al.*, 2011). Alcohol consumption reduced substantially from baseline in both intervention and comparator groups (Table 3). The reduction in consumption was greater in the comparator group than in the intervention group. However, the comparator group had a higher consumption at baseline and, despite showing the greater reduction, had a higher consumption at follow-up. More men in the comparator group reduced their number of drinking days, whereas more men in the intervention group reduced their number of binge drinking days (>8 units in one session). The average weight of participants did not change between baseline and follow-up. Over this period some men lost weight, some remained unchanged and some gained weight.

**Table 3.** Follow-up drinking history and BMI of participants by treatment group

Factor	Control group, *N* = 30	Intervention group, *N* = 31
Hazardous drinkers (positive FAST) (*n*, %)	27 (90.0)	24 (77.4)
Mean weekly consumption (mean units, SD)	38.4 (35.3)	30.8 (33.0)
Mean number of drinking days in previous 28 days (mean, SD)	11.8 (7.9)	13.2 (6.9)
Mean number of binge drinking days in previous 28 days (> 8 units in one session) (mean, SD)	8.4 (6.8)	6.3 (5.8)
Mean BMI (mean, SD)	35.2 (4.0)	35.9^a^ (5.5)

^a^Based on 29 men who attended the final face to face session.

## DISCUSSION

This study identified a group of men at very high risk of developing liver disease, who would greatly benefit from reducing alcohol consumption and losing weight. Overall, this feasibility study has shown that all of the component parts of a randomized controlled trial were completed. Men were recruited; they were willing to engage with an intervention designed to reduce alcohol consumption through the motivation of weight loss; they were retained in the study at follow-up; they were satisfied with the study procedures and the intervention itself; and the outcome data were collected. Process evaluation showed a high fidelity of delivery of the intervention and revealed that the men responded as intended to key steps in the behaviour change strategy. Although no prespecified criteria were set to assess progression to a full trial, the feasibility study has demonstrated that such a trial could be successfully conducted.

This study has provided an estimate of the variance of alcohol consumption which could be used in a sample size calculation for a full trial. It did not attempt to estimate the treatment effect of the intervention. Methodologists recommend against such a practice (Lancaster *et al.*, 2004; Leon *et al.*, 2011) because feasibility studies are underpowered and are vulnerable to imbalance between groups at baseline. In the present study there was such an imbalance. Although mean consumption was lower in the intervention group at follow-up, this cannot be interpreted as meaningful because consumption at baseline was much higher in the comparator group than the intervention group. In fact, a substantial reduction in alcohol consumption occurred in both intervention and comparator groups. This is commonly seen in trials of ABIs (Bernstein *et al.*, 2010; Jenkins *et al.*, 2009). This study used an active control, a conventional brief intervention, so a fall in the comparator group would be expected. These factors will need to be taken into account when estimating the sample size calculation for the full trial.

The recruitment methods exceeded the recruitment target. The two methods produced samples of men who were very similar in demographic characteristics, and alcohol consumption and BMI were slightly lower in men recruited by TSS (data available from the authors). However, recruitment by TSS is much more labour intensive as it involves researchers making many trips to community venues to meet potential participants. GP registers identified a large pool of potential participants who could be contacted by telephone, so recruitment by this method would be recommended for a full trial.

A high rate of follow-up (98%) was achieved. Rates of retention are often much lower in ABI trials (Kaner *et al.*, 2007) and are generally poor in obesity trials (Douketis *et al.*, 2005; Yoong *et al.*, 2013). Several strategies to promote retention were put in place in this study (Brueton *et al.*, 2014). Sessions were organized to be convenient for participants, both in location and timing. Previous studies have shown that men prefer a friendly, relaxed and non-directive style (Gray *et al.*, 2013), with easily understood information (Robertson *et al.*, 2014). The sessions were run in a friendly supportive manner and participants’ questions and concerns were fully addressed. Several methods of contact (mobile phone, home phone, postal address and email address) were used to maximize follow-up. Text messages were sent to keep in touch during the follow-up period. Finally the research team made considerable efforts to arrange follow-up appointments at times and venues convenient for participants. All these techniques should be used in a full trial.

Assessment of fidelity of delivery is particularly important for novel behaviour change strategies (Craig *et al.*, 2008; Miller and Rollnick, 2014). This should determine whether the intervention was delivered as intended and whether participants understood and engaged with the information given (Carroll *et al.*, 2007; Gearing *et al.*, 2011). The face to face sessions used audio recordings to evaluate adherence and competence in the delivery of the intervention. This established that the quality of delivery was high, although there were a few areas where improvement could be made. Fidelity of delivery of text messages was also high. Almost all of the text messages (98%) were delivered to participants’ phones. The nature of the responses to the text messages showed that the men had understood them. The responses also showed that men engaged as intended with key steps in the behaviour change sequence. These approaches illustrate a major role of feasibility/pilot studies; to test whether the intervention functions as intended and to identify areas which need improvement.

A limitation of this study was the loss of ~10% of men between randomization and attendance at the face to face session. It occurred because of difficulties in arranging the session, either because men could not be contacted or they subsequently declined to participate. This loss could be reduced if the time and venue for the appointment were agreed when informed consent had been obtained.

Another possible weakness of this study is that the time period over which the text messages were delivered, 8 weeks, could be too short to promote maintenance of reduced drinking. In particular more time could be allocated to relapse recovery and identifying the benefits of moderated drinking. Another change that should be considered for the full trial would be to lower the entry criterion from >21 units to >14 units of alcohol per week, in line with the new guidelines from the UK Chief Medical Officers (Department of Health, 2015).

## CONCLUSION

The study recruited men at very high risk of liver disease. These men engaged enthusiastically with an intervention intended to change their drinking behaviour through the motivation of weight loss. Further they responded as intended to the key steps in the behaviour change strategy. These findings show that a full randomized controlled trial could be conducted to test the effectiveness of the intervention. There is an urgent need for an intervention to reduce, the very high risk of liver disease in this group of men.
